# Beyond global comparability: why universal health coverage indicators must reflect national contexts

**DOI:** 10.7189/jogh.16.03032

**Published:** 2026-09-18

**Authors:** Eri Osawa

**Affiliations:** National Institute of Public Health, Wako-Shi, Saitama, Japan

**Keywords:** universal health coverage, UHC service coverage index, global comparability, national context, national health indicators, monitoring and evaluation

## Abstract

Universal health coverage (UHC) is a central objective of the global health agenda, supported by internationally standardised indicators that facilitate monitoring and accountability across countries. Although standardised global UHC indicators, such as the Sustainable Development Goals (SDGs), UHC service coverage index, and out-of-pocket healthcare expenditures, support international accountability and collaboration, they do not fully capture domestic healthcare complexities driven by demographic ageing, epidemiological transitions, and evolving health system challenges. These limitations are particularly evident in countries facing complex population health needs. This viewpoint argues that UHC measurements should be adapted to national contexts, considering demographic characteristics, epidemiological profiles, healthcare delivery and financing structures, and political priorities. National governments, in cooperation with international organisations, must adopt a two-tiered monitoring approach that combines global and country-specific indicators and is underpinned by scientifically rigorous data collection. Such a multilayered framework can strengthen both domestic governance and international efforts to achieve UHC.

Universal Health Coverage (UHC) ensures access to the full range of quality health services individuals need without financial hardship [1]. Since the adoption of the United Nations (UN) Sustainable Development Goals (SDGs) in 2015, UHC has become central in the global health agenda, with internationally standardised indicators enabling countries to monitor progress and ensure accountability. The introduction of the global UHC monitoring framework under the SDGs, with harmonised indicators such as the UHC service coverage index (SCI) and out-of-pocket healthcare expenditures, has strengthened global advocacy for UHC and fostered international collaboration towards improving public health. The UHC indicators enabled consistent monitoring across countries, facilitated international benchmarking, and reinforced accountability by providing a common framework for assessing progress [2].

The UHC SCI is a composite indicator that monitors UHC progress globally and across countries [3,4]. The global average UHC SCI score rose from 51 in 2000 to 71 in 2023 [5]. However, given the improvements in service coverage since 2000, as well as differences in demographic composition, epidemiological transitions, and evolving health system challenges and global changes (*e.g.* migration and climate change), a critical question has emerged: ‘Can internationally comparable indicators alone provide sufficient information for national policymaking to achieve UHC beyond the SDGs?’ [6]. Global UHC indicators should prioritise standardisation and conciseness to enable global comparability. However, these limited indicators inherently constrain the ability to fully capture aspects of healthcare system performance that are critical for domestic policymaking. Consequently, even when global reporting suggests steady progress toward UHC, significant challenges remain unresolved at the national level [7].

This viewpoint argues that adapting UHC indicators to national contexts, including demographic and epidemiological profiles, health service delivery and financing structures, and political priorities, is a policy imperative rather than a mere technical add-on. Developing indicators that reflect national circumstances should not be viewed as compromising international comparability. Rather, they can complement global measures by helping countries identify their specific health needs and inform appropriate policy responses. A multilayered approach to UHC monitoring that combines global accountability with national policy relevance could help countries align measurement with their health policy priorities.

## GLOBAL UHC SCI INDICATOR: ESSENTIAL BUT NOT SUFFICIENT

The UHC SCI, jointly developed by the World Health Organization and the World Bank, is a key indicator for evaluating progress toward UHC. It quantifies access to health services on a scale of 0 to 100 under the UN SDGs as Indicator 3.8.1, comprising 14 tracked indicators across four domains: reproductive, newborn, maternal, and child health; infectious disease control; non-communicable diseases (NCDs); and health service capacity and access [4]. This indicator visualises global progress toward UHC and enables international comparisons. Tracking indicators and measurement methods are continuously updated to reflect changing healthcare needs and improved data availability. The 2025 revision updated three indicators: family planning; diabetes treatment coverage; and the healthcare workforce. The UHC monitoring report acknowledges that these indicators do not capture all health services, including mental health, accident, injury, and emergency care [5]. Additionally, they do not reflect quality-related aspects such as equity, quality of care, responsiveness, patient experience, and continuity of care. Although the indicators could be improved, developing applicable indicators for more than 190 countries is challenging, and existing indicators therefore remain the primary basis for global UHC monitoring. However, relying solely on internationally standardised indicators risks confining policy discussions to easily measurable data while overlooking elements critical to healthcare system performance and equity [8].

## WHY THE NATIONAL CONTEXT IS NOW MORE IMPORTANT THAN EVER

Many countries are experiencing accelerated population ageing, a rising burden of NCDs, and increasingly complex care needs that extend beyond one-off healthcare service usage [9]. Alongside service coverage, the quality, effectiveness, and continuity of care, long-term care, and unmet health needs throughout the life course have become critical dimensions of UHC [10–13]; however, these dimensions are not captured by the current UHC SCI. In countries with advanced population ageing, such as Japan, key policy themes include addressing the needs of older adults with multiple chronic conditions, ensuring smooth transitions between medical- and community-based integrated care, and responding to the unmet needs of those with functional limitations [14]. This underscores the growing importance of context-specific approaches to UHC measurement. Our argument is not that the current UHC SCI has led to inappropriate policy decisions. Rather, as countries face increasingly diverse demographic, epidemiological, and health system challenges, globally harmonised indicators alone may be insufficient to guide context-specific policymaking.

## TOWARD A TWO-TIER APPROACH TO UNIVERSAL HEALTH COVERAGE MEASUREMENT

The introduction of indicators that reflect each country’s circumstances should not be viewed as a burden. Rather, UHC measurement should be understood as serving two complementary purposes. Global indicators play a vital role in providing a common framework for international accountability, advocacy, and cross-country comparison. Meanwhile, indicators tailored to national contexts provide the more detailed, context-specific, and timely information required for domestic policy implementation, priority setting, and health system improvement. National context-specific indicators should be developed through a transparent and evidence-informed process, and countries should identify priority health challenges by reviewing national health policies, demographic and epidemiological trends, and burden-of-disease data. Indicators should be selected based on policy relevance, scientific validity, feasibility, and the availability of routinely collected, disaggregated data to monitor equitable access to health services. Measures of out-of-pocket expenditure exceeding a defined threshold should also be included. Finally, these measures could be aggregated into a composite index using an approach similar to that of the current UHC SCI. In short, target values would be defined for each indicator, performance converted to a score from 0 to 100, and scores across service categories combined using a target population-weighted geometric mean to produce a single index [4]. While indicator selection would depend on each country’s policies and data availability, this approach could facilitate consistency with international benchmarks. This two-tiered approach answers different questions. Global indicators track whether countries are moving toward the international UHC goal in a broad sense, whereas country-specific indicators reveal where, for whom, and why progress is insufficient. Recognising this distinction moves the discussion beyond the false dichotomy of choosing between comparability and policy relevance, favouring a practical measurement approach that supports both global accountability and domestic governance. This proposal already exists; for example, several countries have independently developed and calculated their own UHC indicators using their data to reveal their specific trajectories and policy directions. China, for instance, developed a UHC index using 19 indicators, including geographical access to health facilities, health insurance coverage, health personnel, and health expenditure to assess progress between 2002 and 2018 [15]. Malawi similarly combined 23 indicators, including access to water and sanitation and essential medicines, to evaluate UHC [16]. Similar reports have emerged from Myanmar and Kenya [17,18]. The Philippines has likewise established a health sector strategy plan with indicators that complement SDG indicators 3.8.1 and 3.8.2 [19]. Thus, initiatives that guide the evaluation and formulation of health policies using visualised indicators that account for each country’s demographic structure, disease burden, healthcare system(s), and data availability are essential for attaining UHC. Establishing country-specific UHC monitoring systems requires sustained investment in robust health information systems, regular data collection, and analytical capabilities, which remains particularly challenging for many low- and middle-income countries. National governments should proactively monitor health service performance, and international organisations should provide technical and financial support to strengthen national capacity for data collection, analysis, and methodological development. Academic institutions could contribute by developing and validating robust methodological approaches for UHC monitoring, while civil society and other stakeholders could help ensure that UHC monitoring, progress assessment, and future policy directions adequately address national contexts and population needs.

## FUTURE DIRECTION

For UHC measurement to move beyond symbolic reporting and support substantive progress, it must be more closely aligned with national policy priorities and the realities of healthcare systems. Indicators should inform policy decisions about which areas and services require investment, which population groups are being left behind, and where improvements are needed, rather than simply demonstrating progress towards international targets. The proposed framework has not yet been empirically evaluated. Future research should assess whether countries using UHC indicator systems that account for national circumstances are better able to respond to changing health needs and policy priorities than those relying solely on globally standardised indicators. Such evaluations should also examine whether these approaches improve health system performance, equity, and population health outcomes. Additionally, the considerations discussed here primarily concern service coverage and should not be interpreted as diminishing the importance of financial protection, which remains a core component of UHC. Future research should explore whether indicators developed for national use can also strengthen the monitoring of financial protection. Global indicators continue to serve as a common reference for accountability and international comparison, while country-specific indicators could generate the actionable insights required for policy implementation and institutional improvement. Positioning UHC measurement as a multilayered endeavour represents a practical approach that transcends the false dichotomy between comparability and policy relevance. By recognising the complementary roles of global and country-specific indicators, the global health community could strengthen both domestic policymaking and international efforts to achieve UHC.

## Data Availability

**Data availability:** Not applicable.

## References

[1] World Health Organization. Universal health coverage. 2026. Available: https://www.who.int/health-topics/universal-health-coverage#tab=tab_1. Accessed: 27 March 2026.

[2] World Health Organization. World health statistics 2025: monitoring health for the SDGs, Sustainable Development Goals. Geneva, Switzerland: World Health Organization; 2025. Available: https://www.who.int/publications/i/item/9789240110496. Accessed: 8 September 2026.

[3] Hogan DR Stevens GA Hosseinpoor AR Boerma T Monitoring universal health coverage within the Sustainable Development Goals: development and baseline data for an index of essential health services. Lancet Glob Health. 2018;6:e152–68. 10.1016/S2214-109X(17)30472-2 29248365

[4] Department of Economic and Social Affairs. SDGs Indicators Metadata repository. 2026. Available: https://unstats.un.org/sdgs/metadata/. Accessed: 1 April 2026.

[5] World Health Organization. International Bank for Reconstruction and Development / The World Bank. Tracking Universal Health Coverage 2025 Global Monitoring Report. Geneva, Switzerland: World Health Organization and International Bank for Reconstruction and Development / The World Bank; 2025. Available: https://www.who.int/publications/i/item/9789240117808. Accessed: 8 September 2026.

[6] Namyalo PK Wodnik BK Michaelides O Kane S Essue B Ruggerio ED Identifying implementation science research and policy priorities to advance universal health coverage: a multi-country modified Delphi study. BMJ Glob Health. 2025;10:e018562. 10.1136/bmjgh-2024-018562 40780834 PMC12336608

[7] Thomas-McLean H Mak R Noonan CM Yip W Global progress toward universal health coverage: learning from successes and failures. Annu Rev Public Health. 2026;47:459–77. 10.1146/annurev-publhealth-100824-103712 41401513

[8] Cuevas Barron G Koonin J Akselrod S Fogstad H Karema C Ditiu L Universal health coverage is a matter of equity, rights, and justice. Lancet Glob Health. 2023;11:e1335–6. 10.1016/S2214-109X(23)00317-0 37429304

[9] Institute for Health Metrics and Evaluation. Global Burden of Disease 2023: Findings from the GBD 2023 Study. Seattle, WA. USA: IHME; 2025. Available: https://www.healthdata.org/research-analysis/library/global-burden-disease-2023-findings-gbd-2023-study. Accessed: 8 September 2026.

[10] Yanful B Kirubarajan A Bhatia D Mishra S Allin S Ruggerio ED Quality of care in the context of universal health coverage: a scoping review. Health Res Policy Syst. 2023;21:21. 10.1186/s12961-022-00957-5 36959608 PMC10035485

[11] Ranjan A Thiagarajan S Garg S Measurement of unmet healthcare needs to assess progress on universal health coverage - exploring a novel approach based on household surveys. BMC Health Serv Res. 2023;23:525. 10.1186/s12913-023-09542-0 37221549 PMC10207793

[12] GBD 2019 Healthcare Access and Quality Collaborators Assessing performance of the Healthcare Access and Quality Index, overall and by select age groups, for 204 countries and territories, 1990-2019: a systematic analysis from the Global Burden of Disease Study 2019. Lancet Glob Health. 2022;10:e1715–43. 10.1016/S2214-109X(22)00429-6 36209761 PMC9666426

[13] Kowal P Corso B Anindya K Andrade FCD Giang TL Guitierrez MTC Prevalence of unmet health care need in older adults in 83 countries: measuring progressing towards universal health coverage in the context of global population ageing. Popul Health Metr. 2023;21:15. 10.1186/s12963-023-00308-8 37715182 PMC10503154

[14] Katori T Japan’s healthcare delivery system: From its historical evolution to the challenges of a super-aged society. Glob Health Med. 2024;6:6–12. 10.35772/ghm.2023.01121 38450110 PMC10912799

[15] Liu X Wang Z Zhang H Meng Q Measuring and evaluating progress towards Universal Health Coverage in China. J Glob Health. 2021;11:08005. 10.7189/jogh.11.08005 33981413 PMC8088770

[16] Mchenga M Manthalu G Chingwanda A Chirwa E Developing Malawi’s universal health coverage index. Front Health Serv. 2022;1:786186. 10.3389/frhs.2021.786186 36926481 PMC10012749

[17] Han SM Rahman MM Rahman MS Swe KT Palmer M Sakamoto H Progress towards universal health coverage in Myanmar: a national and subnational assessment. Lancet Glob Health. 2018;6:e989–97. 10.1016/S2214-109X(18)30318-8 30056050

[18] Barasa E Nguhiu P McIntyre D Measuring progress towards Sustainable Development Goal 3.8 on universal health coverage in Kenya. BMJ Glob Health. 2018;3:e000904. 10.1136/bmjgh-2018-000904 29989036 PMC6035501

[19] Department of Health. National Objectives for Health Philippines 2023–2028. Manila, Philippines: Department of Health; 2023. Available: https://sites.google.com/view/doh-hfdb/2023-updates/dc-2023-0563?pli=1&authuser=0. Accessed: 9 September 2026.

